# Make Home Pleasant

**Published:** 1864-12

**Authors:** 


### MAKE HOME PLEASANT.

We always suspect errors in domestic training, when we find children preferring all other places to home. Parents should throw around the home circle a magnetism to be found nowhere else. A pleasant and loved home is one of the most powerful restraints from vice, and keeps alive within the heart pure thoughts and generous aspirations. We find some good thoughts on this point in *Life Illustrated:*

"A child may as easily be led to associate pleasure with home ideas as to think of it in connection with the home of his playmates. Certainly, if allowed to do so, he can as readily connect happiness with parents, brothers, and sisters, as with those of other kin. And the child will do so unless happiness and pleasure, when he calls for them under the parental roof, respond 'Not at home!' All home pictures should be bright ones. The domestic hearth should be clean and joyous.

"If home life is well ordered, the children having, according to age, work-time, play-time, books, games, and household sympathies, they will love home, and find pleasure there.

"Give the little ones slates and pencils, and encourage their attempts to make pictures. Drawing will amuse them when noisy plays have lost their zest, or are unseasonable; and the art will be useful to them in all the business of after life. Have them read to each other stories and paragraphs of your selection, and save the funny things and the pleasant ones you see in papers and books, to read to them at your leisure. You cannot imagine how much it will please them, and how it will bind them to you. But choose well for them, for the impression made on their minds now will last when the hills crumble. Have them sing together, and sing with them, teaching them songs and hymns. Let them sing all day, like the birds, at all proper times. Have them mutually interested in the same things, amusements, and occupations; having specified times for each so that their habits will be orderly. Let them work together—knitting and sewing—both boys and girls. They enjoy it equally unless the boys are taught that it is unmanly to understand girls' work. They should know how to do it, and practically, too, as thereby they may avoid much discomfort in future life. Let them work together in the garden—boys and girls—both need out-of-door work. Together let them enjoy their games, riddles, etc.; all their plays, books, and work, while the parents' eyes direct and sympathize, and their voices blend in loving accord. Have the children do some little things, daily, for your personal comfort; let them see that it gives you pleasure, and that you depend on them for the service. This will attach them to you more strongly; and if they feel responsibility, even in matters of themselves trivial, and are sure of your sympathy, their affections and joys will cluster around the home hearth.

"Children like to be useful—it makes them happy. So give them work-time as well as play-time. But in any case, and in all cases, give them sympathy. Express love for them."

---

More than building showy mansion,
More than dress and fine array,
More than domes or lofty steeples,
More than station, power, and sway,
Make your home both neat and tasteful,
Bright and pleasant, always fair,
Where each heart shall rest contented,
Grateful for each beauty there.

More than lofty, swelling titles,
More than fashion's luring glare,
More than mammon's gilded honors,
More than thought can well compare,
See that home is made attractive
By surroundings pure and bright,
Trees arranged with taste and order,
Flowers, with all their sweet delight.

Seek to make your home most lovely:
Let it be a smiling spot,
Where, in sweet contentment resting,
Care and sorrow are forgot;
Where the flowers and trees are waving
Birds will sing their sweetest songs,
Where the purest thoughts will linger,
Confidence and love belongs.

Make your home a little Eden;
Imitate her smiling bowers;
Let a neat and simple cottage
Stand among bright trees and flowers.

There, what fragrance and what brightness
Will each blooming rose display,
Here a simple vine-clad arbor
Brightens through each summer day.

There each heart will rest contented,
Seldom wishing far to roam,
Or, if roaming, still will cherish
Mem'ries of that pleasant home;
Such a home makes man the better,
Pure and lasting its control;
Home with pure and bright surroundings
Leaves its impress on the soul. ANON.

